# Update on Perioperative Ischemic Optic Neuropathy Associated With Non-ophthalmic Surgery

**DOI:** 10.3389/fneur.2018.00557

**Published:** 2018-07-10

**Authors:** Steven Roth, Heather E. Moss

**Affiliations:** ^1^Department of Anesthesiology, and Ophthalmology and Visual Sciences, University of Illinois at Chicago, Chicago, IL, United States; ^2^Departments of Ophthalmology and Neurology & Neurological Sciences, Stanford University, Palo Alto, CA, United States

**Keywords:** cardiac surgery, ischemic optic neuropathy, spinal fusion, optic nerve, anterior ischemic optic neuropathy, posterior ischemic optic neuropathy

## Abstract

Perioperative visual loss (POVL) is a rare, serious complication of non-ophthalmic surgeries. Ischemic optic neuropathy (ION), and retinal arterial occlusion (RAO) are the main causes (1, 2). Less frequent are cortical blindness (3), acute glaucoma (4), and choroidal and vitreous hemorrhage (5). ION is the most common cause for which the neurologist or neuro-ophthalmologist is consulted as it is associated either with a normal ophthalmic exam (posterior ION, PION), or less often, with optic nerve (ON) head swelling (anterior ION, AION). The presumed cause is impaired blood supply to the optic nerve (Figure 1). The most common surgical procedures complicated by ION are cardiac surgery and spinal fusion. Retrospective studies, surveys, and case reports are the basis of most knowledge regarding peri-operative ION (poION), with cohort and case-control studies helping to identify candidate risk factors (6, 7). Animal models have provided insight regarding mechanisms (8). This mini-review is an update on the latest advancements regarding poION in non-ophthalmic surgeries in epidemiological, clinical, and animal studies.

## Epidemiology

Encouragingly, but for uncertain reasons, yearly rates of poION in spinal fusion have decreased, with an overall rate of 0.01% in 1998–2012 (7). Explanations may include greater utilization of minimally invasive spine surgery and surgical staging, and changes in anesthesia practice such as higher blood pressure levels and lower fluid administration (7, 9). Often not appreciated is that poION occurs with cardiac surgery at a rate of 0.06–0.113%, 6-fold greater vs. spinal fusion (6, 10, 11). poION has also been reported after head and neck surgery (12, 13), joint replacement (14), nasal and sinus surgery (15), vascular and general surgery, radical prostatectomy, gynecologic surgery, and liposuction (16).

## Clinical presentation

Symptoms are typically reported within 1–2 d after surgery and frequently upon awakening (17), although first report may be delayed in those sedated after surgery (18). Painless central or peripheral vision loss or both, are common, with color vision decreased or absent, and usually bilateral. Unilateral or asymmetric cases have a relative afferent pupillary defect. Ophthalmoscopic exam does not identify causes for the vision loss other than optic neuropathy. PoION can be AION, with, acutely, a swollen ON head, or PION, with a normal ON head in the acute state; most spinal fusion cases have been PION (16). Lingering anesthetic effects, or sedation, may render patient cooperation difficult for eye examination.

Buono and Foroozan's retrospective series of 83 cases provides insight into poPION's heterogeneity (16), with 54% after spine surgery, 13% followed radical neck dissection, and 33% other surgery. Mean age was 52 years with 66% male. In 75%, visual loss was noted within 24 h. Over 60% were bilateral. Initial visual acuity was light perception in 54% of eyes.

MRI is generally obtained in patients with perioperative visual loss (POVL) to rule out intracranial pathology, and orbital MRI should be obtained to examine the ONs. Usually there are normal retro-bulbar ONs, although there are reports of ON enlargement from edema and perineural enhancement (19, 20). Diffusion-weighted imaging may enhance diagnostic sensitivity (21). Visual evoked potentials (VEPs) are abnormal (22).

Over weeks to months, ON swelling resolves in AION and optic atrophy develops in AION and PION. In Buono and Foroozan's case series, vision improved in 38%, but of 14 with no light perception, 12 (85%) had no improvement (16). A smaller series that included multiple causes of POVL after spine surgery (22/37 ION) reported no change in 68% and worsening in 3% (23).

## Risk factors

Given lack of effective treatments or spontaneous recovery there is substantial interest in risk stratification (e.g., based on pre-existing conditions), and prevention (e.g., through modifying surgical or anesthesia practices). This risk stratification is of importance to patients, who prefer to be informed of the risk of visual loss (24). Though there are no prospective studies, case series, case-control studies, and medical claims-based studies have identified candidate risk factors. To date, no risk prediction model or stratification have been reported.

### Extrapolation from spontaneous NAION

One strategy to identify risk factors is extrapolation from the spontaneous correlate to poION, non-arteritic AION. NAION risk factors include a small optic disk characterized by a small cup-to-disk ratio (C/D), with resulting axonal crowding predisposing to injury. Medical claims-based big data studies have identified association between male sex, white race, and diabetes with end organ involvement and NAION (25).

Medications including amiodarone and phosphodiesterase-5 inhibitors prescribed for pulmonary hypertension and erectile dysfunction are associated with NAION (26). While there are no reports of poION where the drugs were taken within 24 h of surgery, physicians may want to inquire in high risk patients. There is evolving literature supporting obstructive sleep apnea (OSA) as a risk factor for and untreated OSA as a risk factor for second eye involvement of spontaneous NAION (27). A challenge in such extrapolations is that most concern spontaneous NAION, whereas >50% of poION is PION. It remains an unstudied area of research if risk factors for spontaneous and perioperative ION are comparable.

### Potential risk factors for poION in spine surgery

Multiple clinical studies have attempted to identify pre-existing conditions and intra-operative factors associated with poION in spine surgery (For summary, see Table 1). Case series have been striking for capturing the range of perioperative features. A literature based study described lowest hemoglobin 5.8–14.2 g/dL (mean 9.5 g/dL), intraoperative blood loss 0.8–16 L (mean 3.7 L), operative duration 3.5–23 h (mean 8.7 h), and lowest systolic blood pressure (SBP) 48–120 mm Hg (mean 77 mm Hg) in 83 cases (16). To address limitations in case series, The American Society of Anesthesia (ASA) POVL Registry systematically collected reports with detailed anesthesia and surgical data (28). The 93 cases (83 poION and 10 RAO) had mean lowest hematocrit 26% and mean blood loss 2.0 L. Most underwent surgery >6 h, often repeat and multilevel procedures. In 33%, lowest SBP was >90 mm Hg while in 20%, lowest recorded SBP was ≤ 80 mm Hg. Blood pressure decrease from pre-operative baseline varied widely [the definition of “baseline blood pressure” in anesthetic practice remains controversial (30)] with the majority (57%) having SBP or mean arterial blood pressure (MAP) 20–39% below baseline, and 25% SBP or MAP 40–49% below baseline. Deliberate hypotension was used to decrease blood loss in 25%. Median crystalloid fluid administration was 10 L. Surgical positioning devices included the Wilson frame (30%) and Jackson spinal table (27%). Mean age was 50 y. Pre-existing hypertension was in 41%, diabetes 16%, and coronary artery disease 10%. Another series of 37 cases included 8 AION and 14 PION. Less than half had each of hypertension, diabetes, vascular disease, and smoking history. No known vascular risk factors were in 13 (42%). Comparison of 28 of these cases with matched controls found longer operative time and blood loss, but no difference in age, hematocrit or blood pressure (23).

**Table 1:** Summary of the studies on spine fusion and ION mentioned in this review.

**References**	**Type**	**Study size**	**ION #**	**Findings**
Buono and Foroozon (16)	Literature based review	83 (only ION) Lumbar spine	83	*Mean hemoglobin 9.5 g/dl *Mean blood loss 3.7 L *Mean operative duration 8.7 h *Mean lowest SBP 77 mm Hg
Lee (28)	ASA POVL Registry (anonymous case submissions)	93 Lumbar spine	83	*Lowest mean hematocrit 26% *Mean blood loss 2.0 L. *Operative duration mostly > 6 h *33%, lowest SBP was > 90 mm Hg *20%, lowest recorded SBP was *≤* 80 mm Hg. *57% had SBP or MAP 20–39% below baseline *25% SBP or MAP 40–49% below baseline. *Deliberate hypotension in 25%. *Median crystalloid fluid 10 L. *Surgical positioning: Wilson frame 30% and Jackson spinal table 27%. *Mean age 50. *Hypertension 41%, diabetes 16%, and coronary artery disease 10%.
Myers (23)	Case control single institution	37 Lumbar spine	22	*Age, lowest hematocrit and lowest blood pressure no different in cases vs. controls * < 50% hypertension, diabetes, vascular disease, and smoking history. * No known vascular risk factors in 42%.
Lee (18)	Case control multi-institutional	395 Lumbar spine	80	See Table 2 for main findings
Patil (29)	Case control using NIS	600,000 lumbar fusion (also studied cervical spine)	About 120	*Incidence about 0.02% *Increased odds ratio for ION with: Peripheral vascular disease, Diabetes, Hypertension, Obesity, Anemia, Blood transfusion, Hypotension
Rubin (7)	Case control using NIS	2.5M lumbar fusion	257	*Incidence about 0.01% *Increased odds ratio for ION with: age, transfusion, and obesity. Female sex was protective

*ION, ischemic optic neuropathy; MAP, mean arterial blood pressure; SBP, systolic arterial blood pressure*.

To address the limitation of lacking a comparison group, the ASA-POVL Group conducted a case-control study using the Registry and randomly selected, matched controls from 17 academic US and Canadian medical centers (18). By multivariable regression, the six factors associated with poION were male sex, obesity, Wilson frame use, anesthesia duration, large blood loss, and low colloid:crystalloid fluid ratio (Table 2). Limitations are that affected cases were not randomly obtained; rather by anonymous case submissions, of which only a small percentage were confirmed by direct examination of anesthesia records, and missing data. Additionally, it could be argued that the controls are not a random sample of patients undergoing spine fusion, as all were derived only from academic medical centers.

**Table 2:** Factors increasing the odds ratio of developing perioperative ion in lumbar spine fusion surgery.

	**Odds Ratio**	***P* Value**
Male	2.53 (1.35–4.91)	0.005
Obesity	2.83 (1.52–5.39)	0.001
Wilson frame	4.30 (2.13–8.75)	< 0.001
Anesthesia duration, per hour	1.39 (1.22–1.58)	< 0.001
Estimated blood loss, per 1 L	1.34 (1.13–1.61)	0.001
Colloid as percent of non-blood replacement, per 5%	0.67 (0.52–0.82)	< 0.001

*The risk factors were determined using a multivariable analysis in a case-control study. Details are in reference #(18)*.

The Nationwide Inpatient Sample (NIS), a random sample of discharges of 20% of US hospitals, offers advantages of larger sample sizes and less selection bias than the ASA-POVL Registry, but is limited by reduced perioperative data. Analysis from 1993 to 2002 identified hypotension, peripheral vascular disease, and anemia as potential poION risk factors (29). A more recent analysis of >2.5 million discharges with spinal fusion identified older age, male, obesity, and blood transfusion to be associated with poION. These results are important because they were obtained in a very large, randomly collected sample, and suggest the importance of specific pre-operative factors. However, the conclusions rely upon the accuracy of procedure and diagnosis identification based on International Classification of Disease (ICD9) coding with both over- and under-coding possible (31). Also, the definition, timing of (intra- or post-operative), and degree of terms such as hypotension are not specified. Independent confirmation is not possible. A potential application of results from such a population sample is developing risk stratification models applicable to the typical spinal fusion patient.

### Potential risk factors for poION in cardiac surgery

A single center, prospective, case-control study of 602 patients undergoing cardiopulmonary bypass (CPB) identified 8 (1.2%) with poION (all AION). CPB time was longer (252 vs. 164 min), minimum hematocrit lower (18 vs. 21%), 24 h postoperative weight gain higher (18 vs. 11%), and more vasoactive drugs were required in poION cases (17). A single center, retrospective, case-control study of 28,000 patients from 1976–1994 included 17 poION cases (0.06%). PION and AION were not distinguished. PoION cases had lower minimum post-operative hemoglobin and longer CPB. Other associations included clinically severe vascular disease and transfusion. There were no differences in pre- or post-CPB SBP. C/D < 0.3 was in 5 (29%) of poION (10). There was no multivariable model to help interpret interactions between the large number of parameters collected. Holy reported similar results, but included other surgical procedures, complicating interpretation with respect to cardiac surgery (32). All of these studies are limited by small size and single institution design.

A study of >5 million cardiac surgery discharges in NIS between 1998 and 2013, found 794 (0.014%) poION cases. In a multivariable model, poION was associated with male sex, carotid artery stenosis, stroke, diabetic, or hypertensive retinopathy, macular degeneration, glaucoma, and cataract. Cataract was included as a surrogate marker for eye examination to address the concern of under-coding with respect to eye conditions in discharges without poION. Including cataract adjusts model estimates for the confounding variable of eye exam. Therefore, demonstration of positive associations in the models that adjust for cataract increases confidence that other eye diseases are true associations. Intriguingly, this suggests that degenerative eye diseases are associated with poION, raising the possibility of a role of local pre-existing disease in the ON, and the possibility of constructing risk models based on these conditions (6). Limitations are, in addition to those for claims data in general, that type of poION was not identified.

## Mechanism

### Insight from spontaneous NAION

There is considerable literature with respect to spontaneously developing NAION. Delayed filling of the prelaminar optic disk in 76% of NAION eyes and not in normals suggests the filling defect is the primary process, not disk edema (33). Early disruption of the blood-brain barrier in AION, with dye leakage in the ON head (34), correlates with early onset of optic disk edema, even before symptoms (35). A generally accepted theory is that an initial insult leads to optic disc edema and secondary injury to neighboring cells.

**Figure 1:**
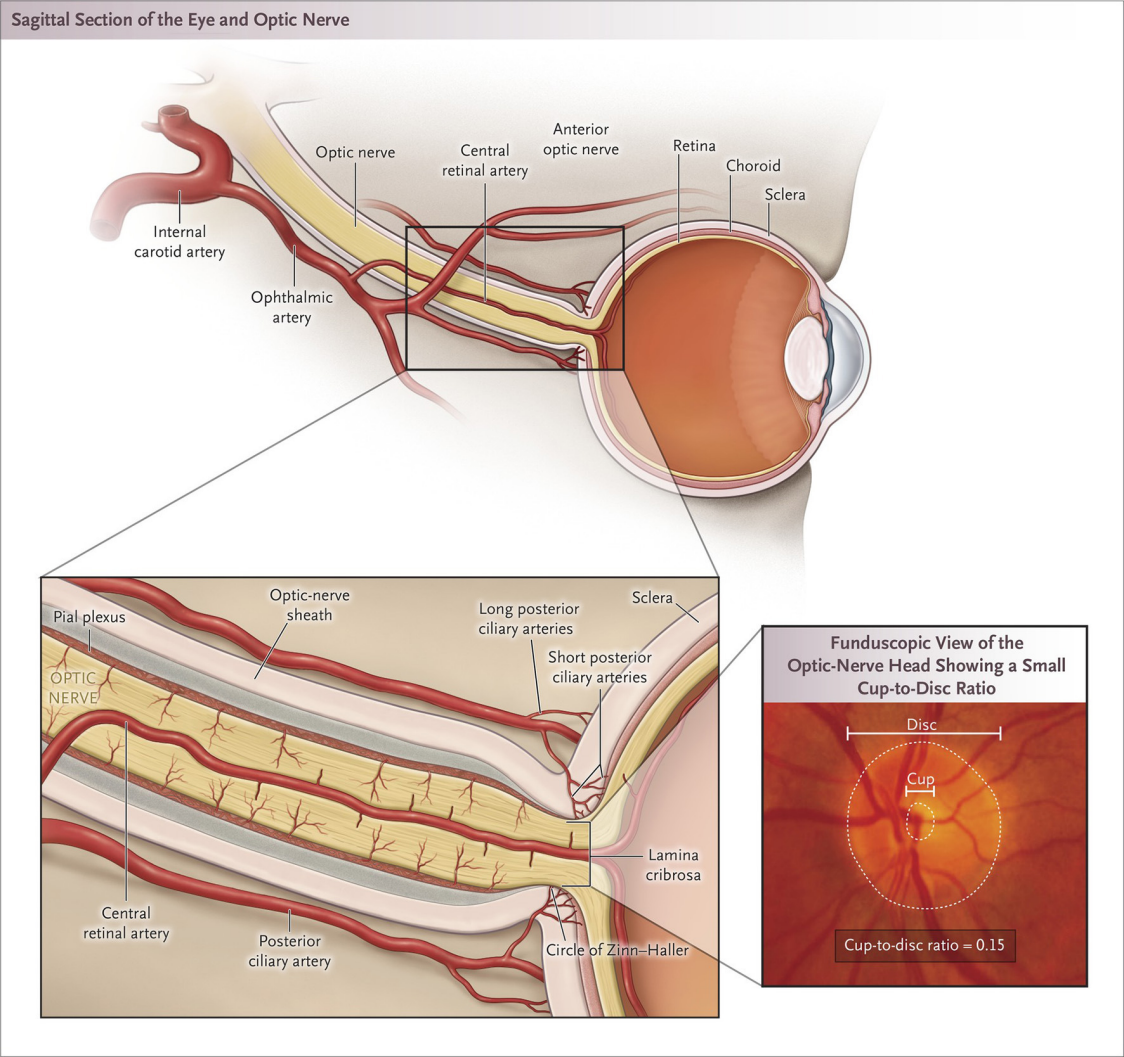
Blood supply to the optic nerve and ON head structure. The blood supply is primarily from the ophthalmic artery. The posterior ON is supplied by pial branches of the ophthalmic artery posteriorly and the posterior ciliary arteries anteriorly; the supply to the central region is limited to the branches that penetrate deeply. The ON head is supplied by the circle of Zinn, from the short posterior ciliary arteries, and from choroidal vessels. The cup-to-disc ratio is the diameter of the central cup divided by diameter of the entire disc [Biousse and Newman (2) used with permission].

Hayreh attributed NAION to individual variations in ON blood supply and watershed areas (36). This theory is supported by anatomic studies and variability of NAION's visual loss. But, inconsistent is that delayed filling of watershed zones was more common in normal eyes than in NAION (34). It has therefore been proposed that reduced perfusion pressure in the region of the para-optic branches of the short posterior ciliary arteries (SPCAs, Figure 1) results in optic disk hypoperfusion, rather than a watershed event (37). Histopathologic examination showed that the infarction was mainly retrolaminar, implicating the SPCAs as the cause of the ischemia (38). Studies in healthy humans generally show preserved anterior ON head blood flow within physiological or lower ranges of perfusion pressure, suggesting that impaired autoregulation may play a role (39–41).

### Insight from histologic examination of peri-operative and spontaneous PION

Histology has been reported in three PION cases after surgical procedures. All showed infarcts in the intra-orbital ON, two with lesions in the axial center and peripheral axonal sparing. The third had peripheral lesions with central axonal sparing in one eye and complete axonal loss in the other (16). It is possible that the location of ischemia varies between cases due to differences is vascular anatomy (42) but this theory requires further study.

A series of 12 cases of posterior ON ischemia in an autopsy study of the ON and chiasm from 53 patients with cerebrovascular disease divided their findings into three stages (43). Pre-morbid visual status was not reported. Acute stage had swollen lesions sharply demarcated from healthy tissue with capillary congestion, swollen axons, and myelin sheaths at the infarct periphery. Intermediate stage showed necrosis, and the chronic stage liquefaction and scar. Similar to poION, location of the ischemic lesions varied with respect to longitudinal and axial location. All had atherosclerotic changes in the intracranial portion of the internal carotid artery and ophthalmic artery, suggesting a role for pre-existing vascular disease.

### Insight from animal studies

The role of proposed inciting intra-operative factors has been investigated in targeted studies. It has been shown in miniature pigs that blood flow in the ON head, as measured by laser Doppler, was maintained during isovolumic hemodilution with hematocrit decreased 30% (44). In a more extreme model, hematocrit at15% and MAP 50 mm Hg significantly reduced total ON blood flow in pigs (45). In the absence of hemodilution, blood flow was preserved in various locations of the optic nerve, including the retrolaminar area, at MAP as low as 40 mm Hg (46). The effects on ON function and histology were not reported in either study. Another limitation is that the blood supply to the ON in pigs differs from humans (47). Taken together, these results demonstrate the robust homeostatic mechanisms for ON head blood supply and suggest multiple perturbations may be necessary to compromise it.

A model that nearly recapitulates some intraoperative events is hemodilution and extreme head down tilt in rats (8), which altered ON function with decreased VEPs and decreased electrical activity (scotopic threshold potential) originating in retinal ganglion cells. Although there was increased ON glial reactivity, frank ischemia was not observed. A rodent model of PION consisting of ischemia induced using photochemical techniques may have application to therapy development, but does not inform understanding of the mechanism of the peri-operative form of the disease (48).

## Treatment

No effective treatment exists. Increasing ocular perfusion pressure pharmacologically or increasing hemoglobin via transfusion may be appropriate when poION is present with hypotension and/or anemia. Both of these interventions as well as hyperbaric oxygen have been associated with improved vision in case reports (16). Diuretics may reduce edema (49), and corticosteroids may reduce axonal swelling, but risk increased wound infection (50). Neither are used widely and have no proven benefit. The use of neuroprotective agents or drugs that lower intraocular pressure with the goal of increasing blood supply to the ON are valuable in theory but have never been shown to result in vision improvement.

## Potential strategies for prevention

Though pre-existing conditions may improve risk stratification, it is operative factors that offer opportunities for direct intervention. The following have received attention due to their theoretical role, and evidence from animal or human studies. None are proven to reduce risk of perioperative ION.

### Length of surgery

Myers (23) and the ASA-POVL Study Group (18) reported increased risk with lengthy spinal fusion surgery. Increasingly, minimally invasive spine surgery is used (51) which reduces operative time, blood loss, and fluid requirements. Another strategy is staging, which trades off shorter individual procedures against multiple surgeries with associated increased risks of infection, and spinal instability, with studies to date still too small to reach definitive conclusions (52–56).

### Avoiding hypotension

Intraoperative hypotension was reported in a majority of cases in one case series and one analysis of the NIS (23, 29). However, it has not been confirmed as a risk factor by case control studies (18, 23, 32). Hypotension is a logical theoretical risk factor (57, 58) that may contribute by decreasing perfusion pressure in ONs that are predisposed due to anatomic variation or abnormal auto-regulation with inability to adequately compensate. What constitutes dangerous hypotension is difficult to quantitate due to lack of data (59). Deliberate hypotension should be viewed as one strategy to decrease blood loss in spine surgery, and factors such as patient's age, pre-existing atherosclerotic disease, hypertension, and its level of control, the possible disadvantages of deeper levels of hypotension (e.g., MAP < 60) vs. any possible advantages, and that it can only decrease arterial bleeding, are among factors that should be considered in its use.

### Minimizing hemodilution and blood loss

Anemia, blood loss, and transfusion have been identified in various clinical studies as associated factors. In uncontrolled hemorrhage without adequate blood volume maintenance, decreased O_2_ delivery to the ON could result in ION (60). Allowing hemoglobin to decrease, as may be done to conserve blood in the operative setting, may be putting patients at increased risk for poION (61). However, level of hemoglobin and duration of decrease that constitute danger to the ON is not known. From the animal studies shown above, caution should be exercised with simultaneous deep levels of hypotension and hemodilution.

### Head positioning

The ASA-POVL case control study showed an association between poION and the Wilson frame use (18). This could be due to venous hypertension. The head may be below the level of the back, in contrast to the Jackson table, where the head is maintained level (62). Positioning effects are evident even in healthy volunteers, who had an increase in ON diameter following prone positioning (63). The patient's head should be in a neutral position during spine surgery, and if a Wilson frame is used, the effect can be achieved with pillows to raise the head, or the bed placed in reverse Trendelenburg (63).

### Fluid administration

ION cases in the ASA-POVL Registry received on average 9.7 L crystalloid intraoperatively (18), and increased postoperative weight gain was identified in a case-control study of visual loss after heart surgery (17), suggesting, although not proving, a role of fluid administration. The odds of developing poION were increased as % colloid of non-blood replacement decreased in spine fusion (18). Fluid resuscitation also contributes to hemodilution. In theory, crystalloid fluids may extravasate from vessels leading to a local compartment syndrome in the ON with edema and compression damaging axons. Colloids may be associated with less edema in the ON, although such an effect has not yet been demonstrated.

### Vasoconstrictors

Shapira showed an association between prolonged epinephrine infusion or long CPB and poION (17). Lee and Lam presented four ION cases in critically ill patients with significant systemic illness and prolonged vasopressors and inotropic agents to maintain blood pressure and cardiac output (64). Based on observation of AION in patients with massive hemorrhage, Hayreh proposed that AION is related to excessive secretion of vasoconstrictors that lowered ON perfusion to low levels (65). However the mechanism is not clear as α-adrenergic receptors are not in the ON and the blood-brain barrier prevents entry of systemically administered agents, except possibly in the prelaminar zone. Therefore, the role of vasopressor use in poION remains unclear.

## Conclusion

Large retrospective studies provided encouraging results that incidence of poION in spinal fusion has significantly declined. Case-control studies have suggested that both pre-operative factors (e.g., pre-existent diseases including pre-existing ophthalmic disease), as well as intraoperative factors such as fluid management, may increase risk of developing perioperative ION in non-ophthalmic surgeries. Although mechanisms of poION in non-ophthalmic surgery remain poorly understood, recent animal studies have provided insight into how proposed contributing factors may act together to cause poION. Diagnostic advances in imaging including diffusion weighted MR may enable early diagnosis.

## Author contributions

All authors listed have made a substantial, direct and intellectual contribution to the work, and approved it for publication.

### Conflict of interest statement

SR has provided expert witness evaluation or testimony in cases of POVL on behalf of hospitals, patients, and physicians. The remaining author declares that the research was conducted in the absence of any commercial or financial relationships that could be construed as a potential conflict of interest.
